# Revaluing donor and recipient bodies in the globalised blood economy: Transitions in public policy on blood safety in the United Kingdom

**DOI:** 10.1177/1363459313476966

**Published:** 2014-01

**Authors:** Helen Busby, Julie Kent, Anne-Maree Farrell

**Affiliations:** University of Leicester, UK; University of the West of England, UK; Monash University, Australia

**Keywords:** biovalue, blood, donors, patients, risk, ‘tissue economies’

## Abstract

The clinical use of blood has a long history, but its apparent stability belies the complexity of contemporary practices in this field. In this article, we explore how the production, supply and deployment of blood products are socially mediated, drawing on theoretical perspectives from recent work on ‘tissue economies’. We highlight the ways in which safety threats in the form of infections that might be transmitted through blood and plasma impact on this tissue economy and how these have led to a revaluation of donor bodies and restructuring of blood economies. Specifically, we consider these themes in relation to the management of recent threats to blood safety in the United Kingdom. We show that the tension between securing the supply of blood and its products and ensuring its safety may give rise to ethical concerns and reshape relations between donor and recipient bodies.

## Introduction: perspectives on blood and tissue economies

Modern biomedicine is dependent upon a range of human tissues and cells, which have applications in transfusion medicine, transplantation medicine and regenerative medicine. Blood has long been used in medicine and continues to have diverse applications in most health-care systems for emergencies and in planned care, hence its description by the World Health Organization (WHO) (2004) as an ‘essential health technology’. Although the use of blood in medicine is an established practice, its apparent stability belies the complexity and change that characterise this field. We aim to explore some of the ways in which the production, supply and deployment of blood has been transformed, drawing on theoretical perspectives from recent work on ‘tissue economies’.

Given the expanded repertoire of uses for human tissues in the 21st century, it has been argued that there is a need to consider the ways in which ‘medical systems that exchange and circulate tissues are also social systems’ (Waldby, 2002: 309). Increasingly, biotechnology has been applied to tissues extracted from humans with the intention of enhancing their applications in the clinic and in research. According to Waldby (2002), ‘Biovalue refers to the yield of vitality produced by the biotechnical reformulation of living processes’ (p. 310). As new forms of organisation have sprung up with a view to mobilising the potential value of these developments, sociologists and anthropologists have explored the fields of enterprise generating biovalue. Here, there has been a particular focus on the novel ways of using human biological materials that have been developed in recent years, their social and economic entanglements and their wider ethical implications – be they embryos, foetuses or other tissues from the living or dead (see, for example, Kent, 2008; Palsson, 2009; Svendsen and Koch, 2008). The prevailing emphasis on new biological materials or innovative applications is in keeping with recent influential accounts of biopolitics and the transformative potential of biosciences (Rose, 2007). Yet, use of blood associated with more established technologies remains highly significant, both in clinical and in economic terms.

As with some newer kinds of tissue donation, ideals of altruism are influential in public policy about blood services and ‘promulgated by professional groups and policy makers’ in these fields (Shaw, 2011: 299). These ideals inform the narrative appeals of organisations involved in soliciting blood (and other tissue) donations (Healy, 2006). While Richard Titmuss’ influential work on gift relationships has been a productive point of reference for thinking about blood services, perspectives from recent work on tissue economies also underline the difficulties in securing separation of human tissues from the commercial realm (Hoeyer, 2009). This difficulty can be understood in the light of a broader argument that objects (including biological objects) often have rather complex lives in the social world, so that ‘the commodity is not one kind of thing rather than another but one phase in the life of some things’ (Appadurai, 1986: 17, cited in Waldby and Mitchell, 2006: 25). Notwithstanding this complexity, principles of voluntariness and altruism weigh heavily in the field of blood donation (ISBT, 2006).

In this article, we argue that while traditional discourses continue to shape practices in some parts of the blood economy, the technical reformulation and division of blood into its component parts and the manipulation of those parts to create new products generates new forms of biovalue. Layered onto these processes are complex and shifting calculations about threats to blood safety, which in turn have implications for relations between donor and recipient bodies. We explore these themes with reference to several key policy issues faced by the blood services in the United Kingdom over the past 10 years. In developing our analysis, we shall draw on materials in the public domain, including documents from blood service organisations and regulators and government policies in the United Kingdom together with reports from international organisations concerned with blood safety and supply, as well as on the scientific and social science literature.^1^

## Deconstructing ‘blood’

The clinical application of blood has a long history. According to the *Oxford English Dictionary*, ‘Blood consists of a mildly alkaline aqueous fluid (plasma) containing red cells (erythrocytes), white cells (leucocytes), and platelets’ (Oxford English Dictionary, 2011). The discovery of the part played by this fluid in maintaining key physiological processes and of the harnessing of blood for use in medical treatments has been well described in the popular book by Douglas Starr, as well as in the professional literature (Giangrande, 2000; Starr, 1998). The first *blood banks* were established in the 1940s, with more systematic services to provide blood to hospitals being set up in the middle part of the 20th century. In the early years of transfusion practice, whole blood was stored and transfused. A reliance on whole blood transfusion changed with the development of fractionating techniques in the United States through the following decades; developments in the 1950s and 1960s eventually allowed for the possibility of manufacturing *products* from plasma proteins in one place, storing them until required and shipping them to where they were needed. Subsequent developments allowed for blood to be broken down into other *components*. These components have specific therapeutic characteristics, and their storage requirements also vary. Over some decades, the use of ‘whole blood’ transfusion has reduced in countries that have resources to process and separate blood (Jersild and Hafner, 2008). Contemporary transfusion medicine is characterised by the use of different components of the blood, administered to patients according to their clinical needs. As well as allowing for better targeted interventions, this allows for each unit of donated blood to be deployed for the treatment of more than one patient. Blood increasingly came to be seen as a scarce resource that needed to be used in the most efficient ways possible. Hence, efficiency is a key principle underlying blood services today.

According to McClelland’s (2007) *Handbook of Transfusion Medicine*, a *blood product* is ‘any therapeutic substance prepared from human blood’ (p. 1). Within this group are two categories: *blood components* and *plasma* (derived) *products*. Blood components may be prepared and administered separately in transfusion as platelets, red cells, white cells, cryoprecipitate and fresh frozen plasma (FFP). Plasma proteins once separated from blood may form the basis for a range of manufactured plasma-derived products. These include immunoglobulin products that have applications in the fields of immunology, neurology, haematology and oncology; albumin that is used to help replace fluid loss after trauma and coagulation factors that are prescribed to alleviate the symptoms of bleeding disorders. Although there have been many technical innovations in processing blood, the long endeavour to produce ‘artificial blood’ with similar functions to blood components for clinical use has not to date been successful.

Scientific and professional discourse distinguishes between *plasma products* and *blood components*. For the sake of clarity, we shall adopt these widely used terms in this article, and the term *blood products* will be used to refer collectively to all therapeutic products derived from blood and plasma. At the same time, we want to unpack the way that the use of these terms has the effect of implying that these are entirely different kinds of material, although they are of course all derived from human blood. In some contexts, two categories of donors have been created: the ‘blood donor’ who donates whole blood and the ‘plasma donor’ who provides plasma only, which is separated from the red blood cells at the point of donation.^2^ This binary categorisation becomes important when we consider that in key supplier countries, ‘plasma donors’ may receive payments (sometimes called compensation), whereas ‘blood donors’ may not.^3^ At the same time, exchange relationships between donor and recipient bodies have become complex, mediated by a network of public and private and non-profit and for-profit institutions.

## Demand in the blood economy

Dynamics of demand and supply in the blood economy have received little attention from those outside the blood services and plasma suppliers. However, we do know that there is a growing global demand for blood and plasma products. Data from the WHO (2004) Global Collaboration on Blood Safety give an indication of the volume of blood donated for therapeutic use in transfusion medicine: some 81 million units of whole blood and 20 million litres of plasma were donated in 2001–2002. More recent data point to an increased demand for plasma for fractionation (O’Mahony and Turner, 2010). Globally, the main applications of whole blood and its components are in the course of surgical procedures, including treatment of trauma patients, and in obstetric care with major bleeding during childbirth (Jersild and Hafner, 2008). Blood is also used in the treatment of medical diseases, especially haematological diseases such as thalassaemia. The use of whole blood, blood components and plasma-derived products does vary considerably across different health systems, with more extensive use being made of a diverse range of blood products in countries with well-resourced health-care systems. Nevertheless, managing periodic or enduring shortages of blood and plasma is a key problem for blood services across the world. Worldwide, people with haemophilia have been the primary users of plasma products, as they are often prescribed coagulation factors on a long-term basis. However, global demand for plasma is today driven by the expansion of immunoglobulin therapies for diverse indications (Farrugia, 2006).

Whereas traditionally blood was thought of in terms of its direct clinical applications, its value is also related to the information that can be derived from it. Once extracted from the body, blood can be used for the purpose of diagnostic tests and for research. Discussion of these wider uses of blood goes beyond the scope of this study, but we want to highlight their close relationship. For example, organisations that obtain blood for use in transfusion or transplant medicine do routinely make it available to research organisations, especially if the blood is in some way ‘surplus’ to requirements or not suitable for human application. As we have seen with the use of other human tissues, the framing of human biological material as surplus or as waste can facilitate the procurement of blood for research uses within a wider blood economy (Kent, 2008; Parry, 2006; Svendsen and Koch, 2008). Whether for research or therapeutic uses, the value of donated blood relates as much to the processes and standards of its procurement and storage as it does to its physical attributes.

## From donor to recipient bodies

Public policies on blood donation in many regions have been shaped by notions of altruism and common good – as well as by the need to provide resources for transfusion medicine (Healy, 2006; Rabinow, 1999; Titmuss, 1970; WHO, 2005). In Britain, the ideals underlying the blood transfusion service chimed with the founding principles of the National Health Service (NHS), launched in 1948: that it should meet the needs of everyone, be free at the point of delivery, and be based on clinical need, not the ability to pay.^4^ The social and ethical importance of voluntary blood donation in Britain was famously expounded by Richard Titmuss (1970), whose work became influential internationally. The importance attributed to voluntary unpaid blood donation was for many years bolstered by a widespread belief that unpaid donation was the primary factor in securing blood safety. With the development of more sophisticated techniques for screening and viral inactivation of blood, safety in blood and plasma is now recognised to be a function of multiple factors, and consequently, the simple equating of safety with unpaid donation provides an inadequate account of contemporary realities. However, the *ideal* of ‘voluntary non-remunerated blood donation’ (VNRBD) remains influential and has become a touchstone for international initiatives on blood services (WHO, 1975, 2005, 2009). Even so, blood donation has diverse meanings and framings in different parts of the world (Adams et al., 2009; Copeman, 2009). Our discussion here will focus on the blood economy in Europe and particularly on recent challenges faced by policymakers and regulators of blood safety in the United Kingdom. We will also refer to policies in the European Union (EU) that impact on blood safety and supply in the United Kingdom, as these are salient in both political and practical terms. We argue that there is increased instability in this area as relations between different actors in this field have become more politicised in recent years.

The association of blood banks in Europe with citizenship, solidarity and imagined national communities continued well beyond the post-war years when many such projects were first established (Rabinow, 1999). Policy on blood sourcing and supply continued to be framed in terms of ethical and political ideals well into the 21st century, as was evident in the discussions surrounding the enactment of the EU ‘Blood Directive’^5^ (Farrell, 2006). Different interpretations of the moral and legal imperatives for VNRBD exist across the EU, as do the local practical arrangements of the various blood services: it is recognised that some organisations operating in the member states do provide some recompense for donors (European Commission, 2011; WHO, 2007). Notwithstanding these diverse practices, the principle of altruism remains very influential in blood policy and law in the EU. Another dimension of the influence of historical ideals in this domain is the suggestion that is sometimes made that blood donation is a ‘universal’ practice – in the sense that all citizens can contribute to the common good by donating blood. In practice, increasingly stringent practices of donor selection and exclusion cut across claims of universalism in this domain (Valentine, 2005). In these and other respects, the ideological framing of blood policy is somewhat in tension with modern practices in this field.

Once the contamination of the blood supply with HIV and Hepatitis C in the 1980s and 1990s was taken up as a matter of public concern, accusations of irresponsibility levelled at health authorities and blood service authorities led to a series of investigations and inquiries in the countries affected (Feldman and Bayer, 1999). Although in the United Kingdom the government declined to take up the call made by patient groups for an official inquiry, an unofficial inquiry was eventually undertaken into these events in England and Wales (Archer et al., 2009). In addition, the Scottish government – having gained substantial devolved powers over health policy in recent years – set up an official inquiry in 2011.^6^ Both inquiries observed the extent to which blood and plasma supply had become more complex than patients had realised, with some products being sourced from outside the United Kingdom. Here as elsewhere, the iatrogenic disasters that impacted on blood services may have shifted public understandings so that blood came to be seen as ‘a distributor not [only] of health and benevolence but of risk and contamination …’ (Waldby and Mitchell, 2006: 52, our brackets).

Following on from widespread recognition of the harm that occurred as a result of patients receiving infected blood in these years, policymakers and regulators have sought to take a precautionary stance to blood safety, seeking to anticipate and manage potential threats (Watkins et al., 2011). Although the threat of transfusion-transmitted HIV or Hepatitis C has greatly receded in countries with well-resourced health systems, the risk of blood-borne infections has become a part of the way that publics and politicians perceive blood. Public recognition of the capacity of blood to transmit feared infections does not replace but sits alongside an understanding of its capacity to sustain and even save lives. Coupled with the value placed on blood donation as ‘in itself’ a social good (*pace* Titmuss), this Janus-faced aspect of blood makes the regulation of safety in this field socially complex and politically salient. Accordingly, those who claim a stake in these decisions include diverse groups going beyond the boundaries of those conventionally involved in patient safety. Blood safety has become a highly politicised issue characterised by litigation, tribunals and inquiries and institutional and regulatory reform (Farrell, 2012).

In recent years, a wider range of interests has mobilised in relation to blood donation as well as blood services. There has been renewed interest on the part of those with an interest in blood donation and supply, those of donors, and of would-be donors who have been excluded, for example, men who have sex with men (Berner, 2011; Hurley, 2009).^7^ There has also been a proliferation of patient groups representing people who are, or expect to be, recipients of plasma products. People with haemophilia, largely marginalised and absent from decision-making in the past, now have organised input into policies on the procurement of blood and plasma products in some national health systems and in the political domain in the EU (O’Mahony and Turner, 2010). Suppliers of plasma products have sought to put forward their perspectives in the public domain, especially on the difficult balance that needs to be struck between safety and sufficiency of supply, and some have developed alliances with patients groups (Plasma Protein Therapeutics Association, 2011).

## Governing the blood supply in Europe

As well as being logistically complex, the supply of blood components and plasma products is politically sensitive in the EU. Two principles that have anchored political and policy discourse about the supply of blood for use in medicine are the ideals of voluntary blood donation already described above and the principle of ‘national self-sufficiency’. As we have seen, the view that blood donation should be voluntary and unremunerated features widely in public policy in many national health systems, as well as in EU law and in international safety initiatives. The principle of ‘self-sufficiency’ refers to the preference of many professionals and politicians for the idea that blood used in a nation’s hospitals should be sourced from donors in the same country. The idea that nations should develop their own blood sourcing and supply arrangements is often traced to a wish to avoid the exploitation of blood donors in developing countries (WHO, 1975). But as Farrugia has observed, policies of ‘self-sufficiency’ have also at times been associated with the belief that some donation systems were more likely to produce safe blood than others, and with assumptions that donors of some nationalities were less desirable than those of others in terms of blood safety: so on the one hand, the deployment of this framework to public policy on the blood supply is resonant of some important ideals and beliefs, but on the other hand, it sometimes has undesirable associations of prejudice and dogma (Farrugia, 2009). In the context of globalisation, notions of ‘self-sufficiency’ are especially problematic and difficult to sustain in relation to the supply of blood and plasma.

The erosion of national ‘self-sufficiency’ of the blood and plasma products supply in the EU context arose from a number of factors that had differential effects on national systems. Among these were the advent of greatly increased demand for blood and plasma and limited investment in the infrastructure for the fractionating of plasma and making of products derived from it. Fractionation and manufacturing techniques were developed that required specialised infrastructure, and new organisations took on this specialised form of production. This infrastructure includes sourcing plasma outside Europe for the manufacture of products distributed worldwide. Once these changes were introduced, plasma products became more stable and acquired a longer shelf life – in contrast to other blood products – and could be more easily shipped to wherever they were required (Starr, 1998). In other words, these properties allowed for the production and the application of plasma products to be decoupled, and this has allowed for the development of specialised organisations and supplier networks for them. As the supply of plasma products became internationalised, a number of specialist plasma producers were established, some of which were not-for-profit organisations and others for-profit companies. This situation contrasts with the supply of blood components, as national blood systems and not-for-profit agencies such as the Red Cross have controlled the supply to a greater extent. However, these not-for-profit blood suppliers fear that arrangements for supplying blood components are liable to change, as entry of for-profit companies into this market threatens the stability and safety of supply that existing providers currently offer (European Blood Alliance, 2009).

So while ‘fresh plasma’ – that is plasma extracted from donated blood, frozen and used within short time scales – continued to be produced by various blood services, the manufacture of products derived from plasma was taken on by specialist producers. For-profit fractionators represent the great majority of fractionating capability worldwide, with not-for-profit agencies accounting for around one-fifth of this activity (Australian Government, 2006). Worldwide, the United States accounts for approximately 70 per cent of plasma collected, and within the United States, the bulk of plasma collected comes from paid plasma donors (Australian Government, 2006: 70). This reliance on US-sourced plasma extends to Europe, including countries that have made extensive efforts to boost their plasma collection capacities. The political sensitivity of relying on paid plasma donors to supply health-care requirements in the EU is such that it is difficult to confirm up to date figures for the plasma economy. However, data from the 1990s point to over 50 per cent of the EU market being sourced from plasma collected in the United States (Farrell, 2006).

It will be evident from this brief outline that the plasma economy does not conform to notions of national self-sufficiency of supply or of unpaid donation but rather it contradicts these. These principles remain influential, however, and accordingly, some countries have sought to retain their own plasma collecting and fractionating capabilities. Nevertheless, the collection and fractionation of plasma and the production of plasma derivatives usually involve considerable interpenetration of private and public agencies and transactions. For example, in the United Kingdom, the company Bio Products Laboratory Limited (BPL) supplies plasma products internationally on a commercial basis, in addition to being a supplier for the NHS. The links between public and private sectors intensified with the purchase by the Department of Health of a US plasma company in 2002, with the aim of securing supply of plasma for the United Kingdom in the wake of alarm about the implications of variant Creutzfeldt–Jakob disease (vCJD) (House of Lords, 2002). Other national plasma providers, such as Sanquin in the Netherlands, also offer contract fractionation services to countries that do not have their own suppliers. In short, the plasma and blood economies are networked and interconnected, characterised by cross border flows of products that are essential to deliver modern health care.

Arrangements for the production of plasma products contrast with the production of blood components – which can be stored for shorter periods of time – for which local or regional supply remains the norm. Blood components are usually supplied from a point relatively close to hospitals where they will be used. In contrast, the production of plasma derivatives is a global business. This state of affairs is sometimes summed up in the phrase ‘blood is national, plasma is global’ (Farrugia, 2009: 125). Thus, there are complex and multiple chains for the supply of a range of products derived from human blood and used in medicine. The procurement and supply of these are shaped to some extent by national governments and regulatory bodies and by coordinating bodies internationally. However, they are also shaped by the global plasma industry. A third factor is that responses to blood safety threats, which are often unpredictable, affect market demand and supply for these products and their value.

## What kind of (bio)value is produced from blood?

As with other tissues and cells, the therapeutic use of blood is mediated through a range of technological interventions. Although blood transfusion originally involved the use of whole blood, as we have seen, it is now more usual for blood to be in the form of components or plasma products. In common with many other biological tissues, the raw material of donated blood requires an investment of effort and resources to be formulated in the ways that are required for its diverse applications (Hoeyer, 2009; Waldby, 2002: 310).

Given the moral sensitivities about discussing blood and its derivatives in the same terms that are used to discuss the prices paid for commodities such as oil, foods or metals, there are some difficulties associated with making an assessment of the monetary value of blood. The approach taken here will be to look at some examples, rather than to endeavour to set out a complete picture of the payments associated with the supply of blood products. As we have seen, there are a range of suppliers for blood and plasma products, some of which are run by national or regional health services and others by separate commercial bodies, not-for-profit agencies or hybrid organisations. As with the sourcing of blood, transactions relating to the use of blood have long been subject to social and ethical constraints. Influential international declarations, such as the WHO’s World Health Assembly Resolution of 1975 and the Melbourne Declaration of 2009, as well as national and international laws and guidelines in many countries prohibit the ‘commodification’ of blood, which may be understood to include the selling of or the making of profit from blood (WHO, 2009). Nevertheless, the processing of blood is associated with significant costs and results in a product that is of value in a clinical context. Subject to logistical and regulatory constraints, these products may be exchanged for a price across different national and regional health systems.

In cases where national health services operate an internal market, as is the case in England, the explicit charging of fees to hospitals for the supply of blood makes it easier to consider this issue. According to National Health Service Blood and Transplant (NHSBT, 2011), which is responsible for supplying blood to hospitals in England and North Wales, the charge to hospitals is currently £125 per unit. As far as we have been able to ascertain, most national blood services do not publish financial statements. However, these services all require specialist infrastructures and staff, and most have systems in place to recoup at least some of the costs of these. In these ways, blood products can acquire exchange value within the systems that supply and use them, notwithstanding the rules prohibiting the buying and selling of blood. Thus, the ideals surrounding voluntary blood donation do not prevent the generation of exchange value from donated blood.

Whereas it is difficult to obtain detailed information on the exchange value of blood products, when it comes to the suppliers of plasma products, more data are available. Healthy sales of plasma products have been reported by commercial plasma fractionators. According to a recent review:

The global market for plasma derived products, and recombinant alternatives, has a combined value of close to US$10.5 billion per annum. The market is dynamic, complex and highly competitive, and in respect of some products there is virtually unrestricted global trade. (Australian Government, 2006: 193)

Some of these products are highly valued for their clinical applications for patients with haemophilia and with rare or orphan diseases. Furthermore, as companies forge alliances with patient groups, they may secure the demand for their products and thereby enhance the monetary value of these goods (CSL Limited, 2009). In addition to advocacy activities by, and on behalf of, a coalition of plasma users (patients) representing the interests of people with these diseases, EU research and development funds targeted at the treatment of rare diseases may facilitate the flow of funds towards these activities (Aymé and Hivert, 2011).

To sum up, the economic dimensions of the extensive trade in plasma are well documented; the availability of data is in part a consequence of the categorising of plasma products as *pharmaceutical* products and of their being supplied by commercial as well as not-for-profit agencies. On the contrary, the monetary value associated with the supply of blood components is not well documented and is difficult to quantify. These activities generally take place within state or non-profit organisations; the language used about the supply of blood by these organisations reflects the prohibition on the buying and selling of blood, the ethics of blood *services*, rather than trade, and is as a result rather opaque. Nevertheless, the supply of blood components is an expensive, complex and resource-intensive activity, with diverse transactions taking place between donors and organisations involved in the procuring, processing and distribution of blood components. While the initial exchange is usually based on voluntary giving of the blood, the component parts subsequently acquire a price or exchange value.

The value of both plasma products and blood components is related to quality management. This in turn depends on demonstrable efforts being in place to screen the donor, donated blood and the inactivation of pathogens.^8^ Even with these measures in place, not all risks can be eliminated. Risks are frequently understood in epidemiological terms as related to donor population. Consequently, if the donor population is known, or assumed, to have a higher rate of any disease transmissible via blood, then the value of blood sourced from that population is likely to be greatly decreased. This is controversial not least because the evidence base for some such exclusion criteria has been subject to criticism, as we have seen, for example, in relation to the exclusion of men who have sex with men from giving blood in blood services in many countries (Grenfell et al., 2011; Hurley, 2009). It has become evident that the social implications of exclusion as a blood donor may be highly problematic for those involved, especially if such policies are perceived to be unjust or not merited.^9^

## Risk and precaution in blood policy in the United Kingdom

Pathogens that have been considered a threat to blood safety over the past quarter century include those associated with HIV, malaria, Chagas disease, West Nile Virus and vCJD. As Farrugia has observed, the capacity of wealthier nations to deal with both established and emerging infections is impressive.^10^ However, ‘the key common feature of the main blood safety threats of the past quarter century has been their unpredictability’ (Farrugia, 2009: 125). In common with a widely used terminology, we shall refer to infections that can be transmitted to recipients of donated blood as transfusion-transmissable infections (TTIs). As well as their unpredictability, the impact of these TTIs on the blood supply is shaped by the dynamics of globalisation including the international mobility of both people and products. Increasingly, new blood service requirements have created categories of people (including sex workers, ‘men who have sex with men’, intravenous drug users and people who have travelled to specified geographic regions) who are not considered to be safe blood and plasma donors due to their being considered at higher risk of having infections. These measures, intended to enhance safety, have at times reduced the availability of blood, making it more difficult to access fresh blood for clinical use, with adverse consequences for patient care (Farrugia, 2009).

An example of the problem of striking the right balance between safety and sufficiency in the blood supply is the situation facing policymakers and regulators in the United Kingdom, where concerns arose that the presence of vCJD in the wider population may be reflected in the blood donor population. Notwithstanding the considerable uncertainty around both the prevalence of the infective agent in the population and its transmission, a series of precautionary measures was put in place following recognition of this potential threat to blood safety (Lefrere and Hewitt, 2009). All the four UK blood services accepted the recommendation by the government’s advisory committee to import FFP from outside the United Kingdom for child patients (aged less than 16 years). FFP for these patients is therefore procured from the United States (Committee on the Safety of Blood, Tissues and Organs, 2012a). The use of UK-sourced plasma was allowed to continue for use for most adult patients, although this too was kept under review. Other measures have included a move to source plasma for the manufacture of plasma derivatives from outside the United Kingdom: BPL Ltd, a supplier of key plasma-derived products for the NHS, has been prohibited from processing plasma sourced from UK donors since 1998. One potential further measure that has been discussed is the possibility of introducing a test for the prion associated with vCJD, known as PrPSc, to the blood screening procedures, if a sufficiently sensitive and reliable test were to become available (Bennett and Dobra, 2009). It is recognised, however, that the interests of donors and of recipients of blood may be somewhat different in relation to such a test: while stringent screening is seen to be in the interests of patients, it may not be in the interests of donors to receive knowledge of a reactive test since interpretation of the implications of such a test is difficult at present and there is no known effective treatment for vCJD (Franklin, 2004). As well as leading to the ‘deferral’ (exclusion) of donors whose blood tested positive PrPSc, it is anticipated that some regular blood donors would choose not to continue if such a test were introduced (Bennett and Dobra, 2009).^11^ According to the UK governments’ advisory committee, recent data suggest that early estimates of prevalence of vCJD in the UK population may have been overly pessimistic (Committee on the Safety of Blood, Tissues and Organs, 2012b). Therefore, it was announced that while precautionary measures already implemented are being kept in place, some further measures that had been under active consideration would not be implemented at this time. Meanwhile, one wider consequence of these developments has been that people from the United Kingdom, or who have lived in the United Kingdom for significant periods, are prohibited from donating blood and plasma in many countries.

Although the incidence of vCJD in the population has presented a particular challenge to blood services in the United Kingdom, the policy response to this exemplifies the broader transition that has taken place from traditional blood banking primarily within national boundaries to international networks for the procurement and supply of blood products described above. First, the paradigm of national self-sufficiency in blood has been eroded by the difficulties that many countries face in achieving a national supply, including logistical constraints, resource limitations and safety considerations. Second, there is a tension between the stated ideal of altruistic (unpaid) donation and the reality that the main global suppliers of plasma products pay donors in order to secure sufficient supplies of plasma. A third point brought out by these developments is that the assumed commonality of interests between donors and patients does not always hold true; at some critical points in the trajectory of the blood services, significant tensions may arise between the interests of donors and patients. Fourth, despite the dichotomy of blood components and plasma products, the common source of these in human blood means that ‘safety threats’ connect the two categories. Finally, the dilemma about the eventual implementation of tests for blood donors for vCJD infectivity offers an illustration of the trade-off between safety and supply in the blood supply: the imposition of higher safety thresholds, whether in relation to blood screening or the screening of donors, tends to reduce the supply of blood. In this context, the careful management of the limited resource of blood that conforms to a specified safety threshold becomes an important aspect of the tissue economy.

## Conclusion: ethical relations in modern blood economies

Blood services are embedded in a narrative that is distinctly nostalgic, but the social relations between people who donate blood, people who need blood and people who work with blood have been transformed in the past 50 years (Busby, 2010). Despite common assumptions that ‘blood is blood’ – as patients and professionals are reported to have said, referring to the sheer ubiquity and mundaneness of blood in hospital settings (Pfeffer and Laws, 2006) – we have shown that such assumptions do not recognise key features of blood economies. Rather as we have seen blood is a material that is intensively processed, tested, discarded, used in short time or fractionated to be used in multiple products, made available to some patients, though not always in sufficient quantity or quality.

In the wake of the blood scandals of the 1980s, potential safety threats have been at the centre of contemporary public policy and risk management practices. Regulatory reform created a bifurcated approach to the regulation of blood and plasma and has sought to harmonise standards in the industry while also attempting to promote the ethical values that have traditionally shaped blood procurement practices (Farrell, 2009). This tension between the ethics of donation and the imperatives of ensuring a safe supply has had a number of effects, which we have outlined here. Potential threats to the blood supply and scarcity of blood products sometimes follow from interventions designed to reduce exposure to TTIs.

We also suggest that socio-ethical relations between donors and recipients in the blood economy have been transformed and merit closer consideration as do the ways in which ‘consumer’ interests are represented within the sector. The political stakes are high here as the need to protect public health is balanced with industry, professional and public or patient concerns. Donor deferral policies that exclude some groups from donating blood are just one arena where the politics of blood play out particular notions of risk.

Finally, we suggest that social analysis of blood has neglected important aspects of what has increasingly become a global bioeconomy. The production of blood itself – far from being immutable – is both technically and socially complex. The often tacit processes underlying blood sourcing and supply are deserving of continued vigilance and analysis because it will enrich both our understanding of how donor bodies are being revalued in the contemporary bioeconomy and how within this policy arena the tensions between safety and supply shape strategies for the governance of risk.

## Notes

1. This article is informed by an ongoing qualitative study about ‘Risk, safety and consent in blood services in the UK’, which aims to explore the framing and management of risk and safety in relation to blood transfusion by professionals, regulators, patients’ societies and people who receive blood in the United Kingdom. We make particular reference to policy in the United Kingdom and to policy and law on blood safety in the European Union (EU), which strongly influences the UK regulatory environment. While reference is made to overviews of the international dimensions of blood economies, there is much important work about international policy on blood supply and blood safety that we have not been able to reference here, given the constraints of word limits. We have extended the focus on blood services to include some consideration of the supply and use of plasma for fractionation.

2. In apheresis, blood is briefly extracted from the body, but only the plasma is collected for use while the red cells are returned to the donor. Plasma may also be sourced from donated (whole) blood.

3. The donation of blood and plasma is subject to diverse rules in different countries. In the United States, plasma donors may receive monetary compensation. The global plasma industry is highly reliant upon the United States for the supply of source plasma used in the manufacture of plasma derived products (Farrugia et al., 2010).

4. The National Health Service Act 1946 (9 and 10 Geo 6. Ch. 81). London: HMSO.

5. Directive 2002/98/EC of the European Parliament and of the Council of 27 January 2003 setting standards of quality and safety for the collection, testing, processing, storage and distribution of human blood and blood components and amending Directive 2001/83/EC.

6. http://www.penroseinquiry.org.uk/preliminary-report/

7. The Committee for the Safety of Blood, Tissues and Organs in the United Kingdom recently recommended that the policy of excluding men who have sex with men be changed to one of deferral for a fixed period (Hurley, 2011).

8. In this article, we focus on the *supply* of blood products, rather than their administration. It should be noted, however, that the *treatment* context is known to be an important factor in transfusion safety. In the United Kingdom, for example, it is reported that patients are more likely to be put at risk by receiving the wrong blood component than by TTIs (Knowles and Cohen, 2011).

9. We note that policies on donor exclusion are not necessarily contested or controversial: they may be characterised by a high degree of consensus. See Berner’s (2011: 384) consideration of responses to the emerging threat posed by HIV by organisations representing gay men in Sweden in the 1980s.

10. World Health Organization (WHO, 2010) data show that donation rate and extent of viral testing are dependent on national economic status.

11. The blood services in the United Kingdom refer to the turning away of blood donors as ‘deferral’, even if it is intended that they will be permanently excluded from donating blood.
